# Preventing Unnecessary Costs of Drug-Induced Hypoglycemia in Older Adults with Type 2 Diabetes in the United States and Canada

**DOI:** 10.1371/journal.pone.0162951

**Published:** 2016-09-20

**Authors:** Mathieu Boulin, Vakaramoko Diaby, Cara Tannenbaum

**Affiliations:** 1 Institut Universitaire de Gériatrie de Montréal, Quebec, Canada; 2 College of Pharmacy and Pharmaceutical Sciences, Florida A&M University (FAMU), Tallahassee, Florida, United States of America; 3 Université de Montréal, Faculties of Medicine and Pharmacy, Quebec, Canada; University of Colorado Denver School of Medicine, UNITED STATES

## Abstract

**Background:**

The costs of drug-induced hypoglycemia are a critical but often neglected component of value-based arguments to reduce tight glycemic control in older adults with type 2 diabetes.

**Methods:**

An economic (decision-tree) analysis compared rates, costs, quality-adjusted life-years, and incremental costs per quality-adjusted life-year gained associated with mild, moderate and severe hypoglycemic events for 6 glucose-lowering medication classes in type 2 diabetic adults aged 65–79 versus those 80 years and older. The national U.S. (Center for Medicare Services) and Canadian public health payer perspectives were adopted.

**Findings:**

Incidence rates of drug-induced hypoglycemia were the highest for basal insulin and sulfonylureas: 8.64 and 4.32 events per person-year in 65–79 year olds, and 12.06 and 6.03 events per person-year for 80 years and older. In both the U.S. and Canada, metformin dominated sulfonylureas, basal insulin and glucagon-like peptide1 receptor agonists. Relative to sulfonylureas, thiazolidinediones had the lowest incremental cost-effectiveness ratios in the U.S. and dominated sulfonylureas in Canada for adults 80 years and older. Relative to sulfonylureas, dipeptidyl peptidase4 inhibitors were cost-effective for adults 80 years and older in both countries, and for 65–79 year olds in Canada. Annual costs of hypoglycemia for older adults attaining very tight glycemic control with the use of insulin or sulfonylureas were estimated at U.S.$509,214,473 in the U.S. and CAN$65,497,849 in Canada.

**Conclusions:**

Optimizing drug therapy for older type 2 diabetic adults through the avoidance of drug-induced hypoglycemia will dramatically improve patient health while also generating millions of dollars by saving unnecessary medical costs.

## Introduction

Hypoglycemia in older adults living with type 2 diabetes significantly affects quality of life and healthcare expenditures [1–3]. The bulk of costs represent hospital inpatient stays and prescriptions [2,3]. Rates of emergency room visits for hypoglycemia dramatically increase with age, from 9.6 events per 10,000 person-years in 60–69 year olds, to 19.6 events per 10,000 person-years in adults aged 80 years and older [4]. The American Diabetes Association, the American Geriatrics Society, and the Canadian Diabetes Association encourage more flexible hemoglobin A1c targets in older adults depending on the patient’s clinical condition: <7.5%, <8%, and <8.5% for patients with good, complex/intermediate, and very complex/poor health status, respectively [5,6]. The 2014 U.S. National Action Plan for Adverse Drug Event Prevention [7], the 2014 Veteran Affairs hypoglycemic safety initiative [8] as well as the 2015 Choosing Wisely campaign [9] similarly prioritize strategies to curb drug-induced hypoglycemia among older adults.

Approximately 43% of older U.S. adults with diabetes attain very tight glycemic control (hemoglobin A1c level below 6.5%), with almost half using insulin or sulfonylureas [10]. Insulin and sulfonylureas confer a fourfold higher risk of being hospitalized for hypoglycemia in diabetic adults [11], leading to an underestimation of the hidden costs associated with prescribing these agents. There is a clear need to calculate the savings potentially achievable from disinvesting in drugs that promote hypoglycemia, from both the patient and systems-level perspective [12,13]. The main objective of this analysis is to estimate the hypoglycemia-related costs and loss of quality of life associated with the use of 6 classes of glucose-lowering medications in older adults across the U.S and Canada.

## Methods

An economic (decision-tree) analysis compared age-stratified average rates, costs and decrements in quality of life of mild, moderate and severe drug-induced hypoglycemic events associated with the use of 6 classes of glucose-lowering medications. The national U.S. (Center for Medicare Services) and Canadian public health payer perspectives were adopted. The time horizon chosen was a 1-year period to assess the short-term consequences of hypoglycemia. Only direct medical costs were considered for both the U.S. and Canada. Costs and quality-adjusted life-years (QALYs) were not discounted as the temporal framework of the study was one year.

### Modeling

**Fig 1:**
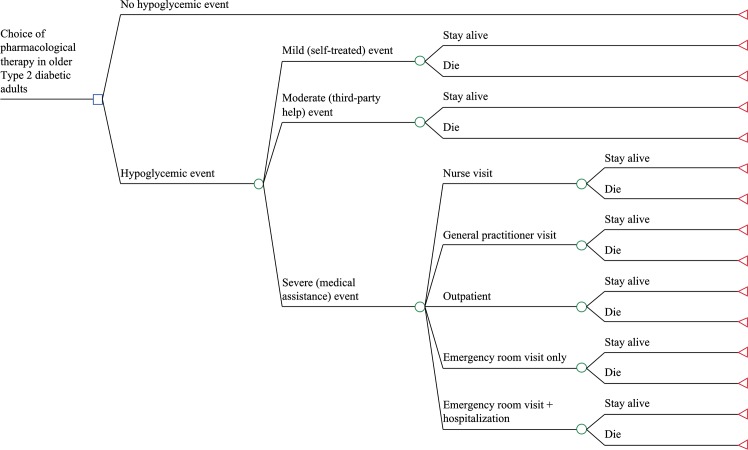
Decision-tree model for drug-induced hypoglycemia in older type 2 diabetic adults. *Square* indicates the decision node (choice of glucose-lowering therapy between metformin, sulfonylurea, dipeptidyl peptidase4 inhibitor, thiazolidinedione, glucagon-like peptide1 receptor agonist, and basal insulin); *circles* indicate chance nodes; *triangles* indicate terminal nodes.

A decision-tree was constructed for older type 2 diabetic adults requiring glucose-lowering therapy, not controlled with diet alone. Mild hypoglycemic events were defined as an episode of hypoglycemia during which no help is required to resolve the situation; moderate events were defined as an episode of hypoglycemia during which the patient requires non-medical third-party assistance (i.e., family members/friends); severe hypoglycemic events were defined as an episode of hypoglycemia during which the patient requires medical assistance [14]. Severe hypoglycemic events may lead the patient to visit a general practitioner or a nurse practitioner (primary care) or he/she may be treated in an outpatient practice, an emergency room or hospitalized with injury, fracture and/or cardiac or neurological complications. Each hypoglycemic event requiring a hospitalization results in the patient staying alive or dying (Fig 1).

In the base-case model, metformin was used as the reference medication. Based on its advantageous efficacy and safety profile, both U.S. and Canadian recommendations consider metformin as first-line pharmacologic therapy in older adults with type 2 diabetes [5,6]. Alternative strategies included: sulfonylureas, dipeptidyl peptidase4 inhibitors, thiazolidinediones, glucagon-like peptide1 receptor agonists, and basal insulin (glargine). The model assumes that the 6 classes of glucose-lowering medications have approximately equivalent efficacy in achieving recommended hemoglobin A1c levels in older type 2 diabetic adults [5,6]. The model was also run with sulfonylureas as a second reference medication. Sulfonylureas rival metformin as the most frequent agents used for treating older type 2 diabetic adults in the US and Canada, prescribed to 30–40% of individuals [10,15,16]. The decision-tree analysis was conducted separately for 65–79 year olds versus 80 years and older [4]. Medication classes were considered separately and not in combination.

The outcomes considered in the analysis were incidence rates of hypoglycemia, annual costs (therapy and hypoglycemia), QALYs, and the incremental costs per QALY gained. We used the definition for a willingness-to-pay threshold based on the per capita gross domestic product [17]. The U.S. per capita gross domestic product in 2014 was U.S.$54,630 [18]. The Canadian per capita gross domestic product in 2014 was CAN$53,891 [18], converted from U.S.$50,271 using an exchange rate of 1.0720 on July 3, 2015.

### Model input parameters

#### Incidence rates of mild, moderate and severe hypoglycemia by medication class and age group

Average rates of mild hypoglycemic events per person-year by age group for each medication class were calculated using a validated model that combines measures of average diabetes duration, hemoglobin A1c, body mass index, and glomerular function rate [19] (Table 1). Average values of hemoglobin A1c, body mass index, and glomerular function rate in diabetic adults aged 65–79 years versus 80 year olds were obtained from the literature [20]. The average type 2 diabetes duration in the two age groups was estimated at 15.6 years for 65 to 79 year olds [21], and 17.8 years for those individuals aged 80 years and older [22].

**Table 1:** Average parameter values for calculation of incidence rates of mild hypoglycemia.

	Age group		Reference
	65–79 years	≥80 years	
**Base rate by medication class (event/person-year)**			
• Metformin	1.0	1.0	[19]
• Sulfonylurea	3.0	3.0	[19]
• Dipeptidyl peptidase4 inhibitor	1.0	1.0	[19]
• Thiazolidinedione	1.0	1.0	[19]
• Glucagon-like peptide1 receptor agonist	1.3	1.3	[19]
• Basal insulin (glargine)	6.3	6.3	[19]
**Type 2 diabetes duration (years)**	15.6	17.8	[21,22]
**Hemoglobin A1c (%, mmol/mol)**	7.1, 54.0	7.0, 53.0	[20]
**Body mass index (kg/m**^**2**^**)**	31.1	28.2	[20]
**Glomerular function rate (ml/mn)**	61.0	45.0	[20]

Incidence rates for mild hypoglycemic events were calculated from the following equation [19]:

Rate of mild hypoglycemia = Base rate by medication class x Risk_Duration_ x Risk_Hemoglobin A1c_ x Risk_Body mass index_ x Risk_Glomerular function rate_

Risk_Duration_ = 1.037^Min[duration,20.0]-9.0years^

Risk_Hemoglobin A1c_ = 0.82^(Hemoglobin A1c-7.0%)^

Risk_Body mass index_ = 0.95^Min[Max[body mass index,21.0]35.0]-33.0^

Risk_Glomerular function rate_ = 22700^(Min[60.0,Max[glomerular renal function,15.0]]-0.86655–60.0–0.86655)^/1.2

Severe hypoglycemic events were predicted to occur at a frequency of 1.25% in the U.S. population [19]. As no robust estimate of moderate hypoglycemic events could be found, we combined moderate and severe hypoglycemic events into a single category with a predicted frequency of 5% [23].

Incidence rates of hypoglycemia by age group and medication class were assumed to be the same in the U.S. and Canada. Although the sulfonylurea drug glipizide is available in the U.S. only, and gliclazide is available in Canada only, the hypoglycemic risk of the two drugs is similar [24]. All other drugs are available in both countries.

#### Probability and type of healthcare resource use

After a mild hypoglycemic event, 14% of diabetic adults contact a primary care provider, and an average of 3.9 additional self-monitoring blood glucose tests (strip and needle) are used [25]. For moderate events, 2% of type 2 diabetic adults receive an injection of glucagon [26]. In the case of a severe hypoglycemic event, we estimated that 26% and 13% will be managed by a general practitioner or nurse practitioner in primary care; 20% in outpatient practice, 17% will be managed in the emergency room, and 24% hospitalized [26, 27]. Only 35% of patients who call an ambulance for hypoglycemia are taken to hospital, 25% of whom receive an injection of glucagon [28]. 85% of type 2 diabetic adults presenting to Canadian emergency departments for hypoglycemic events transported by ambulance [29]. After a severe hypoglycemic event, we assumed an average number of 3.9 additional self-monitoring blood glucose tests and a probability of 14% of general practitioner contacts [25]. The average daily number of blood glucose tests by medication class is: 0.94 for metformin, dipeptidyl peptidase4 inhibitor, thiazolidinedione, and glucagon-like peptide1 receptor agonist users, 1.16 for sulfonylurea users, and 2.08 for basal insulin users [30]. All input parameters including healthcare resources and probabilities for use as well as assumptions driving the economic model are detailed in Table 2.

**Table 2:** Input parameters for the base-case model.

Probabilities or numbers	Values	Assumptions	Reference
**Hypoglycemic event by severity**			
Mild event	0.95	Data from the United Kingdom Hypoglycemia Study Group in 102 sulfonylurea users	[23]
Moderate event	0.04	Data from the United Kingdom Hypoglycemia Study Group in 102 sulfonylurea users	[23]
Severe event	0.01	Data from the Archimedes model simulation with 10,000 U.S. Type 2 diabetic adults over a period of 3 years	[19]
**Fatality**			
Mild or moderate hypoglycemic event	0.00	Assumes 100% of older adults not seeking medical assistance will live	-
Severe hypoglycemic event without hospitalization	0.00	Assumes 100% of older adults not requiring hospitalization will live (including those managed in and released from an emergency room)	-
Severe hypoglycemic event with hospitalization	0.20	1-year mortality rate among 376,617 Medicare beneficiaries hospitalized for hypoglycemia	[3]
**Healthcare resource use**			
*After a hypoglycemic event whatever its severity*			
Healthcare professional contact	0.14	U.S. data from 691 diabetic patients after a mild hypoglycemic event; assumes the same frequency after a moderate or severe hypoglycemic event; assumes healthcare professional contact corresponds to a general practitioner visit for a medium, established patient	[25]
Blood glucose, quantitative assay	0.14	Assumes 1 assay per healthcare professional contact for any hypoglycemic event	[25]
Self-monitoring blood glucose test, average additional number*	3.90	U.S. data from 691 diabetic patients after a mild hypoglycemic event; assuming one strip and one lancet use per test; assumes the same frequency after a moderate or severe hypoglycemic event	[25]
*After a moderate hypoglycemic event*			
Glucagon injection	0.02	Canadian data from 255 diabetic patients requiring third-party help for hypoglycemia	[26]
*After a severe hypoglycemic event*			
General practitioner visit	0.26	U.S. administrative claims database of a southeastern managed care plan; data derived from 2,315 Type 2 diabetic patients	[27]
Nurse practitioner visit	0.13	“	[27]
Outpatient	0.20	“	[27]
Emergency room visit only	0.17	“	[27]
Hospitalization	0.24	“	[27]
Glucagon injection	0.25	Data from 546 diabetic patients with a hypoglycemic event requiring attendance by the emergency medical services in South Central England	[28]
Ambulance use	1.00	“	[28,29]
*Therapy-related*			
Home blood glucose monitor	1.00	Assume 100% of older adults using a home blood glucose monitor regardless the drug	-
Needles*	1.00	One per injection of basal insulin or glucagon-like peptide1 receptor agonists	-
Self-monitoring blood glucose test, average number per day with*			
• Metformin, dipeptidyl peptidase4 inhibitors, thiazolidinediones, and glucagon-like peptide1 receptor agonists	0.94	Utilization study of blood glucose test strips in type 2 diabetic adults in Ontario	[30]
• Sulfonylureas	1.16	“	[30]
• Basal insulin (glargine)	2.08	“	[30]

*Numbers

#### Costs

Direct medical costs retained in the study were those related to healthcare resource use due to hypoglycemic events and glucose-lowering therapy. We used 2015 Centre for Medicare costs and equivalent resource use and cost sources in Canada (S1 Text). Costs are listed in S1 Table for the U.S. and Canada. Costs were represented in 2015 U.S.$ for the U.S. and in Canadian$ (CAN$) for Canada.

#### Utilities

Utility and disutility values were obtained from representative populations in Canada and the U.S. (n = 8,286) [31]. Twelve hypoglycemic health-states associated with diabetes were directly valued using the time trade-off method [32]. The average utility value for uncomplicated diabetes was 0.844 [31]. The estimated average disutility values per hypoglycemic event type (mild and moderate or severe; daytime and nocturnal) per year in the type 2 diabetic population are presented in S2 Table. Daytime versus nighttime mild and moderate/severe hypoglycemic events were predicted to occur in a proportion of 75% and 60% respectively [25,33].

### Sensitivity analyses

One-way deterministic and probabilistic sensitivity analyses were conducted. When the 95% confidence intervals were not available for the parameters, a range variation of ± 25% of the base-case value was applied. Gamma distributions were used for incidence rates of hypoglycemia, costs and disutility values; a beta distribution was used for the utility value for uncomplicated diabetes. A Monte-Carlo simulation was run for 1,000 iterations and cost-effectiveness acceptability curves were created from these analyses. All analyses were performed in Microsoft Excel 2010.

### Population costs of hypoglycemia

The annual cost of drug-induced hypoglycemia was calculated for older U.S. and Canadian type 2 diabetic adults attaining very tight glycemic control (hemoglobin A1c level below 6.5%) with the use of insulin or sulfonylureas, using per person costs derived from our model by age group (65–79 years and 80 years and older). In the U.S., we estimated that 1.2 million older adults with type 2 diabetes attain very tight glycemic control with the use of insulin or sulfonylureas [10,34]. Our calculations assume a prescription ratio of 2.3 (41%/18%) of sulfonylureas/insulin in older type 2 diabetic adults attaining very tight glycemic control in the U.S. [15], and a proportion of octogenarians and older in the U.S. of 26% [35]. In Canada, approximately 1.2 million older adults have type 2 diabetes [36,37]. In the absence of specific Canadian data, we projected U.S. data [10,15] to estimate the number of older type 2 diabetic adults with very tight glycemic control in Canada: 128,626 divided by 38,978 and 89,648 older adults using insulin and sulfonylureas, respectively. In Canada, the proportion of octogenarians and older among all adults aged 65 years is also 26% [36].

We also calculated the annual savings potentially achievable in older type 2 diabetic adults attaining very tight glycemic control from disinvesting in insulin and sulfonylureas for each one of the following three options: *i*) no drug replacement; *ii*) replacement with the most cost-effective agent; and *iii*) replacement with a 50–50% mix of the two most cost-effective agents.

## Results

### Incidence rates, costs and QALYs associated with drug-induced hypoglycemia by medication class and age group

Incidence rates of drug-induced hypoglycemia were highest for basal insulin and oral sulfonylurea agents in 65–79 year olds (8.64 and 4.32 events per person-year, respectively), and even higher for octogenarians (12.06 and 6.03 events per person-year, respectively). Hypoglycemia occurred with an identical low rate in metformin, dipeptidyl peptidase4 inhibitor and thiazolidinedione users: 1.44 versus 2.01 events per person-year in adults aged 65–79 years, and in octogenarians and older, respectively. Metformin was associated with the lowest per-person annual cost of hypoglycemia: U.S.$433 and CAN$309 for 65–79 year olds, and U.S.$472 and CAN$356 for octogenarians (Table 3). In comparison, the per-person annual cost of hypoglycemia in 65–79 year old individuals using sulfonyurea drugs was U.S.$709 and CAN$750, and for insulin was U.S.$1,522, and CAN $2,206 (Table 3). QALYs mirrored rates of drug-induced hypoglycemia, with the highest values for metformin, dipeptidyl peptidase4 inhibitors, and thiazolidinediones, and the lowest values for basal insulin and sulfonylureas. For all medication classes, QALYs were lower for adults aged 80 years and older, reflecting higher rates of drug-induced hypoglycemia.

**Table 3:** Incidence rates, costs and quality-adjusted life-years associated with drug-induced hypoglycemia by medication class.

	Incidence rate of hypoglycemia(events per person-year)*		Annual cost per person†		Quality-adjusted life-years*
	Mild hypoglycemia	Moderate/severe hypoglycemia	U.S.(2015 U.S.$)	Canada(2015 CAN$)	
**Adults aged 65–79 years old**					
Metformin	1.37	0.07	433	309	0.831
Sulfonylureas	4.10	0.21	709	750	0.805
Dipeptidyl peptidase4 inhibitors	1.37	0.07	2,307	1,228	0.831
Thiazolidinediones	1.37	0.07	996	835	0.831
Glucagon-like peptide1 receptor agonists	1.71	0.09	2,332	2,942	0.828
Basal insulin (glargine)	8.21	0.43	1,522	2,206	0.770
**Adults aged 80 years and older**					
Metformin	1.91	0.10	472	356	0.826
Sulfonylureas	5.73	0.30	827	892	0.789
Dipeptidyl peptidase4 inhibitors	1.91	0.10	2,347	1,276	0.826
Thiazolidinediones	1.91	0.10	1,035	883	0.826
Glucagon-like peptide1 receptor agonists	2.39	0.12	2,381	3,002	0.821
Basal insulin (glargine)	11.47	0.60	1,788	2,528	0.735

* Incidence rates of hypoglycemia and quality-adjusted life-years were the same in the U.S. and Canada

†Annual cost per person includes cost of healthcare resources due to hypoglycemic events and cost of glucose-lowering therapy (medications including dispensing fees, testing supplies, and needles for insulin and glucagon-like peptide1 receptor agonists)

### Incremental costs per QALY gained

Table 4 shows the results of the cost-effectiveness analyses. In the U.S. and Canada, metformin was less costly than thiazolidinediones and dipeptidyl peptidase4 inhibitors for both age groups (equivalent QALYs for the three classes of glucose-lowering medications). In the U.S. and Canada, metformin dominated sulfonylureas, glucagon-like peptide1 receptor agonists and basal insulin for both age groups. Deterministic and probabilistic sensitivity analyses confirmed the dominance of metformin over all other classes of glucose-lowering medications (data not shown).

**Table 4:** Results of the cost-effectiveness analyses.

	Incremental cost per quality-adjusted life-year gained			
	U.S. (2015 U.S.$)		Canada (2015 CAN$)	
	65–79 years	≥80 years	65–79 years	≥80 years
**Relative to metformin**	*-*	*-*	*-*	*-*
Thiazolidinediones	Dominated	Dominated	Dominated	Dominated
Dipeptidyl peptidase4 inhibitors	Dominated	Dominated	Dominated	Dominated
Sulfonylureas	Dominated	Dominated	Dominated	Dominated
Glucagon-like peptide1 receptor agonists	Dominated	Dominated	Dominated	Dominated
Basal insulin (glargine)	Dominated	Dominated	Dominated	Dominated
**Relative to sulfonylureas**	-	-	-	-
Metformin	Dominant	Dominant	Dominant	Dominant
Thiazolidinediones	10,988	5,703	3,294	Dominant
Dipeptidyl peptidase4 inhibitors	61,342	41,746	18,378	10,539
Glucagon-like peptide1 receptor agonists	71,193	48,796	96,201	66,244
Basal insulin (glargine)	Dominated	Dominated	Dominated	Dominated

The cost-effectiveness of different classes of glucose-lowering medications are ranked in decreasing order relative to the comparator. Dominated means that the corresponding alternative is more expensive and less effective* than the comparator (either metformin or sulfonylureas); Dominant means that the corresponding alternative is less expensive and more effective than the comparator (sulfonylureas)

*Relative to metformin, thiazolidinediones and dipeptidyl peptidase4 inhibitors are more expensive (same QALYs for the three classes of glucose-lowering medications).

Relative to sulfonylureas, thiazolidinediones had the lowest incremental cost-effectiveness ratios in the U.S., and dominated sulfonylureas in Canada for adults aged 80 years and older. Dipeptidyl peptidase4 inhibitors were shown to be cost-effective relative to sulfonylureas for adults aged 80 years and older in both countries, and for 65–79 year olds only in Canada. Results were robust to deterministic sensitivity analyses with incidence rates of hypoglycemia and medication costs as main drivers of the model (S1 Fig). The Monte-Carlo simulation results did not diverge significantly from the base-case analyses showing that thiazolidinediones were most cost-effective after metformin and that glucagon-like peptide1 receptor agonists and basal insulin had 0% chance of being cost-effective (S2 Fig).

### Population costs of hypoglycemia

Using data from Table 3, the U.S. annual cost of insulin- and sulfonylurea-induced hypoglycemia in older type 2 diabetic adults attaining very tight glycemic control was estimated to be U.S.$232,571,387 and U.S.$276,643,356, respectively, for a combined cost of U.S.$509,214,473. In Canada, the annual cost of insulin- and sulfonylurea-induced hypoglycemia in older type 2 diabetic adults attaining very tight glycemic control was estimated to be CAN$29,913,883 and CAN$35,583,966, respectively, for a combined cost of CAN$65,497,849.

Disinvestment in insulin and sulfonylureas among older type 2 diabetic adults attaining very tight glycemic control in the U.S. would result in potential annual savings of U.S.$ 1,207,222,133, U.S.$ 671,022,734, and U.S.$ 330,407,733 in the case of no drug replacement, 100% replacement with metformin, and 50%/50% replacement with metformin/thiazolidinediones, respectively. In Canada, the corresponding annual savings equal CAN$ 159,793,856, CAN$ 118,476,616, and CAN$ 51,934,925 in the case of no drug replacement, 100% replacement with metformin, and 50%/50% replacement with metformin/thiazolidinediones, respectively (S3 Table).

## Discussion

With respect to cost, quality of life and the number of hypoglycemic events averted, metformin remains the recommended first-line agent in the pharmacologic management of type 2 diabetes in the elderly [5,6,38]. Thiazolidinediones and dipeptidyl peptidase4 inhibitors emerged as agents of choice after metformin. Thiazolidinediones, while advantageous from a hypoglycemia perspective, are associated with a myriad of other safety considerations not accounted for in our analysis. These include fluid retention and congestive heart failure [39], bone fractures [40], and possibly bladder cancer [41]. The European Medicines Agency [42], France [43] and some jurisdictions in the U.S. and Canada [44,45] suspend or restrict marketing authorization for the thiazolidinedione drugs rosiglitazone and pioglitazone, limiting widespread use.

Dipeptidyl peptidase4 inhibitors showed favorable cost-effective profiles for adults aged 80 years and older in both the U.S. and Canada, as well as for adults aged 65–79 in Canada only. However, dipeptidyl peptidase4 inhibitors were not found to be cost-effective in adults aged 65–79 in the U.S. because of higher drug acquisition costs. Glucagon-like peptide1 receptor agonists incurred slightly higher rates of hypoglycemia, and relative to sulfonylureas were only cost-effective in the U.S. for adults aged 80 years and older, with an incremental cost per QALY gained close to U.S.$50,000. The additional disutility associated with injectable treatments in type 2 diabetic patients [46], and in particular with exenatide which requires two injections per day, as well as the risk of nausea and weight loss in older frail adults [47], decreases the attractiveness of the glucagon-like peptide1 receptor agonists as second agents for many individuals. Though widely prescribed, neither basal insulin nor sulfonylureas should be recommended as second-line options if avoidance of drug-induced hypoglycemia is of priority.

Despite compelling evidence about the costs and loss of quality of life associated with drug-induced hypoglycemia in older adults, a substantial proportion of primary care physicians still do not factor these data into clinical care decision-making [48]. The prevalence of insulin and sulfonylurea use has remained relatively stable over time among older diabetic U.S. adults with complex/intermediate or very complex/poor health status attaining a hemoglobin A1c level of less than 7% [10]. As the population ages, clinicians will increasingly be asked to consider the risks, costs and effects on quality of life associated with drug-induced hypoglycemia when choosing glucose-lowering therapy for older type 2 diabetic adults. Our analyses indicate that up to one billion dollars of unnecessary Medicare expenses could be avoided, if more judicious use of glucose-lowering agents were considered.

Strengths of our study include estimating the economic consequences of hypoglycemia by medication class. A two-country comparison reproduced the majority of the findings, except where drug acquisition costs strongly diverged. Several limitations warrant consideration. In the absence of robust data on hypoglycemia in older type 2 diabetic adults treated with 2 or more glucose-lowering agents, only monotherapy was considered in the decision-tree model, providing conservative estimates of cost and hypoglycemia. Over 50% of older U.S. type 2 diabetic adults receive a single glucose-lowering agent in clinical practice [4,15]. Among those receiving 2 or more glucose-lowering agents, metformin is prescribed in approximately 90% of the cases [15], suggesting that the majority of cases of multi-drug therapy for diabetes will follow the same trends observed in our model. We used a recently validated model to tailor prediction of the rate of mild hypoglycemic events in type 2 diabetic adults as a function of disease duration, hemoglobin A1c level, body mass index, kidney function, and class of glucose-lowering medication [19]. The age-stratified mean values obtained may not be generalizable to individual patients with unique risk profiles. Ascertainment of the overall risk of hypoglycemia in our model assumed a constant proportion of mild, moderate and severe hypoglycemic events. Though imperfect, our results are consistent with other reports in the literature [49].

## Conclusions

Metformin is economically advantageous as first-line therapy in older type 2 diabetic adults. Basal insulin and sulfonylureas are not cost-effective agents with respect to hypoglycemic events and associated hypoglycemia-related quality of life. The choice of thiazolidinediones, dipeptidyl peptidase4 inhibitors, and glucagon-like peptide1 receptor agonists require additional clinical considerations, though dipeptidyl peptidase4 inhibitors appear to be a reasonable second choice for patients aged 80 years and older. Optimizing drug therapy for older type 2 diabetics through the avoidance of drug-induced hypoglycemia will dramatically improve patient health while also generating millions of dollars by saving unnecessary medical costs.

## Supporting Information

S1 Fig One-way deterministic sensitivity analyses (Tornado diagrams). (A) For type 2 diabetic adults aged 65–79 years using dipeptidyl peptidase4 inhibitors relative to sulfonylureas in the U.S. The incremental cost per quality-adjusted life-year gained in the base-case was U.S.$61,342. (B) For type 2 diabetic adults aged 80 years and older using dipeptidyl peptidase4 inhibitors relative to sulfonylureas in the U.S. The incremental cost per quality-adjusted life-year gained in the base-case was U.S.$41,746. (C) For type 2 diabetic adults aged 65–79 years using glucagon-like peptide1 receptor agonists relative to sulfonylureas in the U.S. The incremental cost per quality-adjusted life-year gained in the base-case was U.S.$71,193. (D) For type 2 diabetic adults aged 80 years and older using glucagon-like peptide1 receptor agonists relative to sulfonylureas in the U.S. The incremental cost per quality-adjusted life-year gained in the base-case was U.S.$48,796. (E) For type 2 diabetic adults aged 65–79 years using dipeptidyl peptidase4 inhibitors relative to sulfonylureas in Canada. The incremental cost per quality-adjusted life-year gained in the base-case was CAN$18,378. (F) For type 2 diabetic adults aged 80 years and older using dipeptidyl peptidase4 inhibitors relative to sulfonylureas in Canada. The incremental cost per quality-adjusted life-year gained in the base-case was CAN$10,539. (G) For type 2 diabetic adults aged 65–79 years using glucagon-like peptide1 receptor agonists relative to sulfonylureas in Canada. The incremental cost per quality-adjusted life-year gained in the base-case was CAN$96,201.(H) For type 2 diabetic adults aged 80 years and older using glucagon-like peptide1 receptor agonists relative to sulfonylureas in Canada. The incremental cost per quality-adjusted life-year gained in the base-case was CAN$66,244.
(PDF)

S2 Fig Cost-effectiveness acceptability curves. (A) For type 2 diabetic adults aged 65–79 years in the U.S. (B) For type 2 diabetic adults aged 80 years and older in the U.S. (C) For type 2 diabetic adults aged 65–79 years in Canada. (D) For type 2 diabetic adults aged 80 years and older in Canada.
(PDF)

S1 Table Cost of healthcare resource use and glucose-lowering therapy. (DOCX)

S2 Table Utility, disutility and frequency of events data used in the model. (DOCX)

S3 Table Potential annual savings from disinvesting in insulin and sulfonylurea prescriptions in older type 2 diabetic adults attaining very tight glycemic control. (DOCX)

S1 Text Cost of healthcare resource use and glucose-lowering therapy in the United States and Canada. (DOCX)
