# Supplementary figures and images for: Preventing Unnecessary Costs of Drug-Induced Hypoglycemia in Older Adults with Type 2 Diabetes in the United States and Canada
Source: PLoS One. 2016 Sep 20;11(9):e0162951. doi: 10.1371/journal.pone.0162951 (PMC5029920; doi:10.1371/journal.pone.0162951)

A.

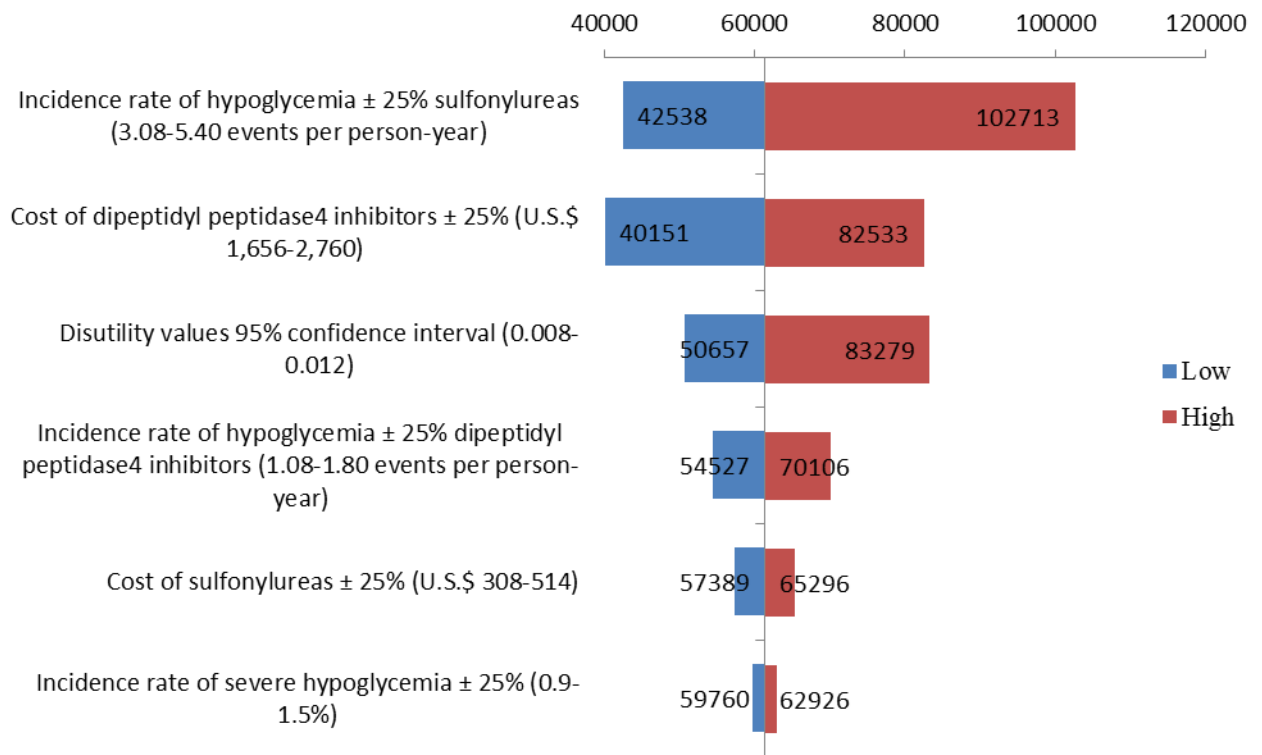

B.

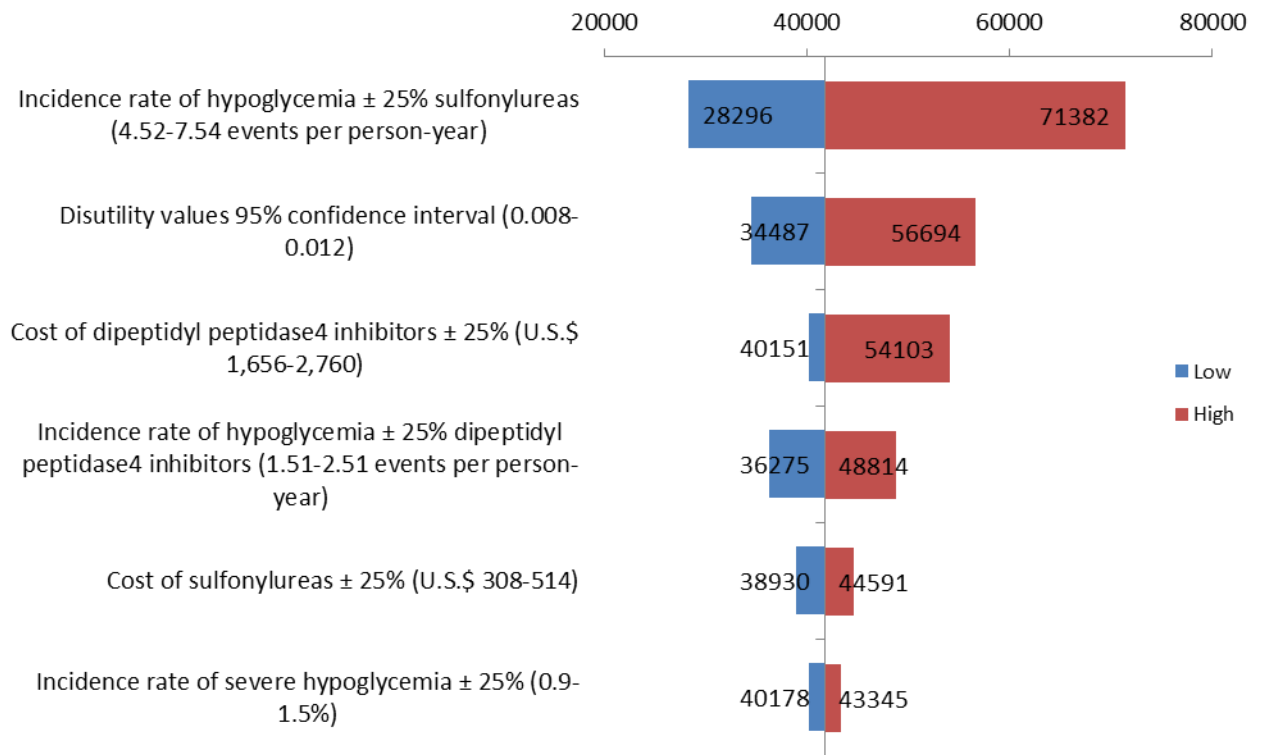

C.

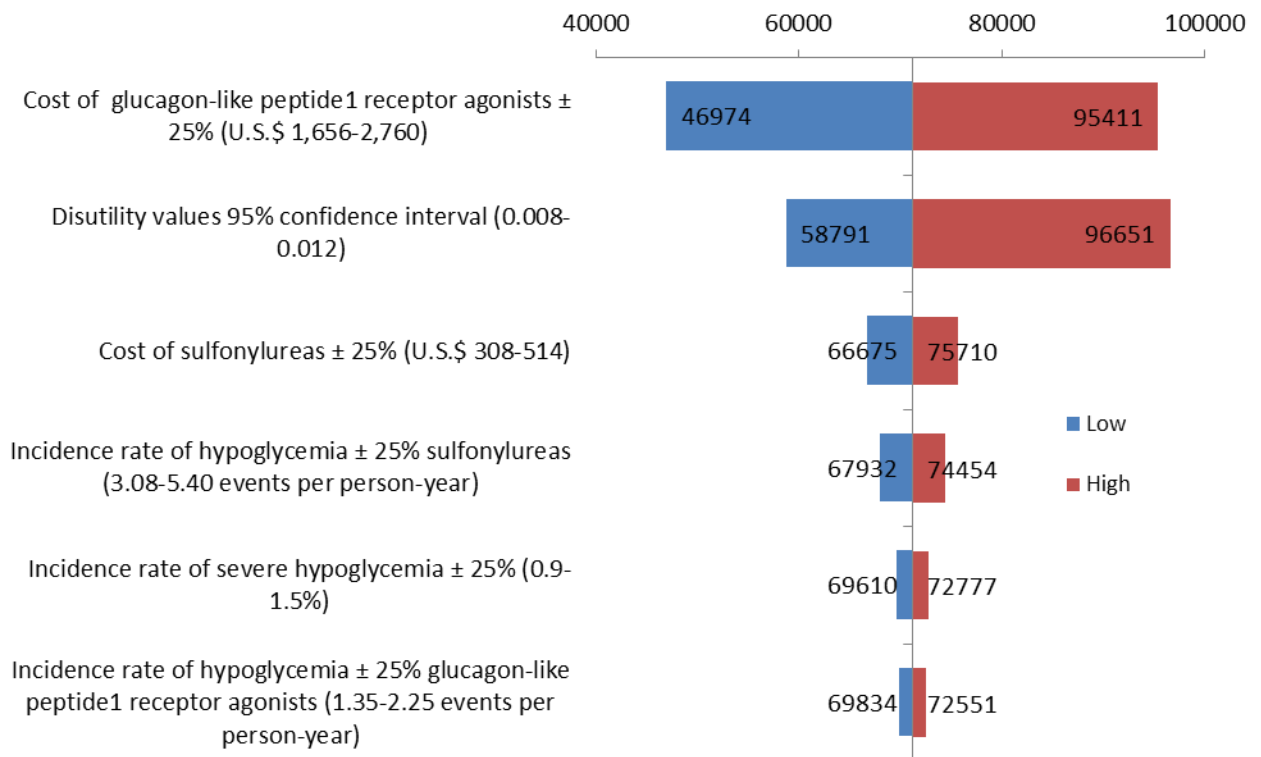

D.

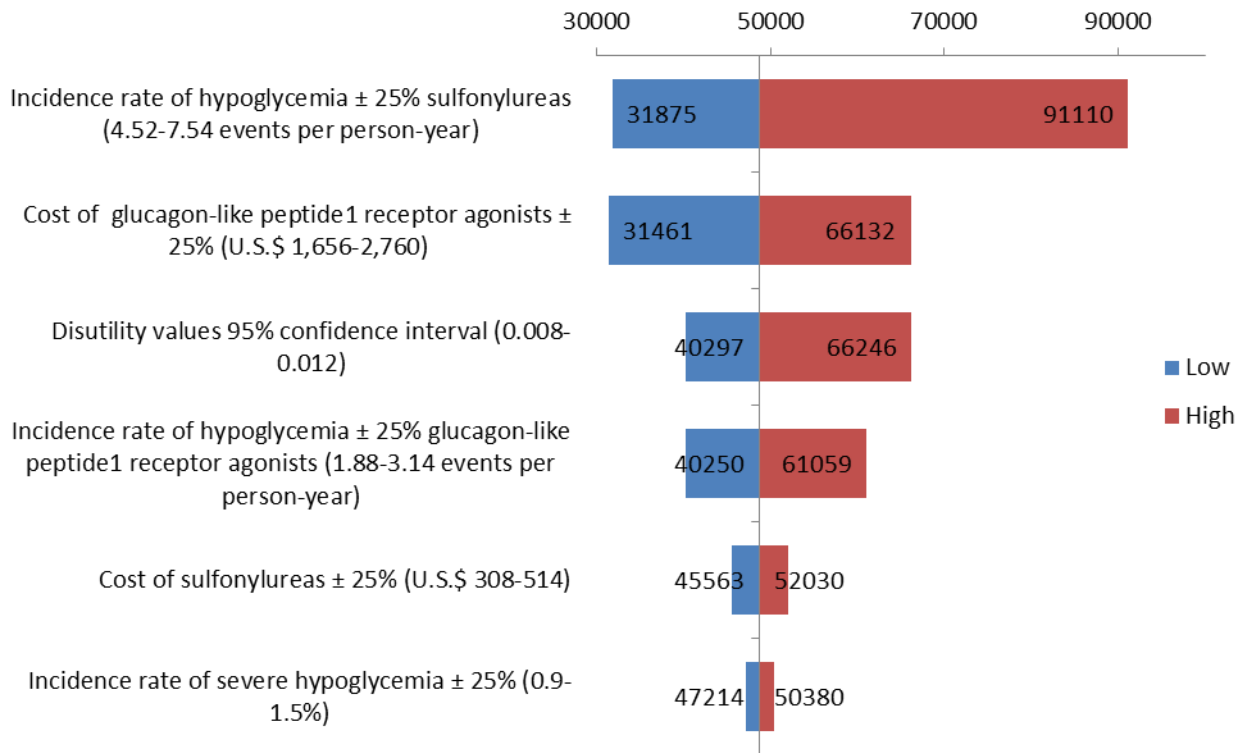

E.

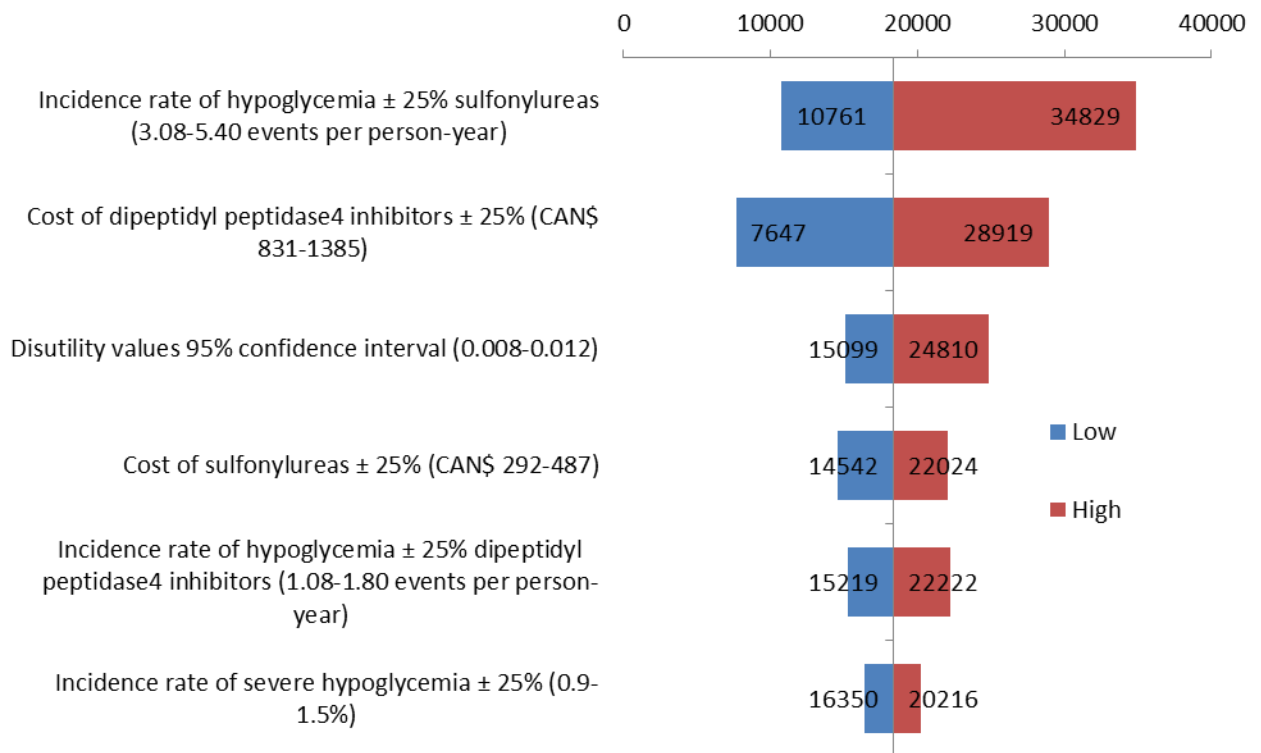

F.

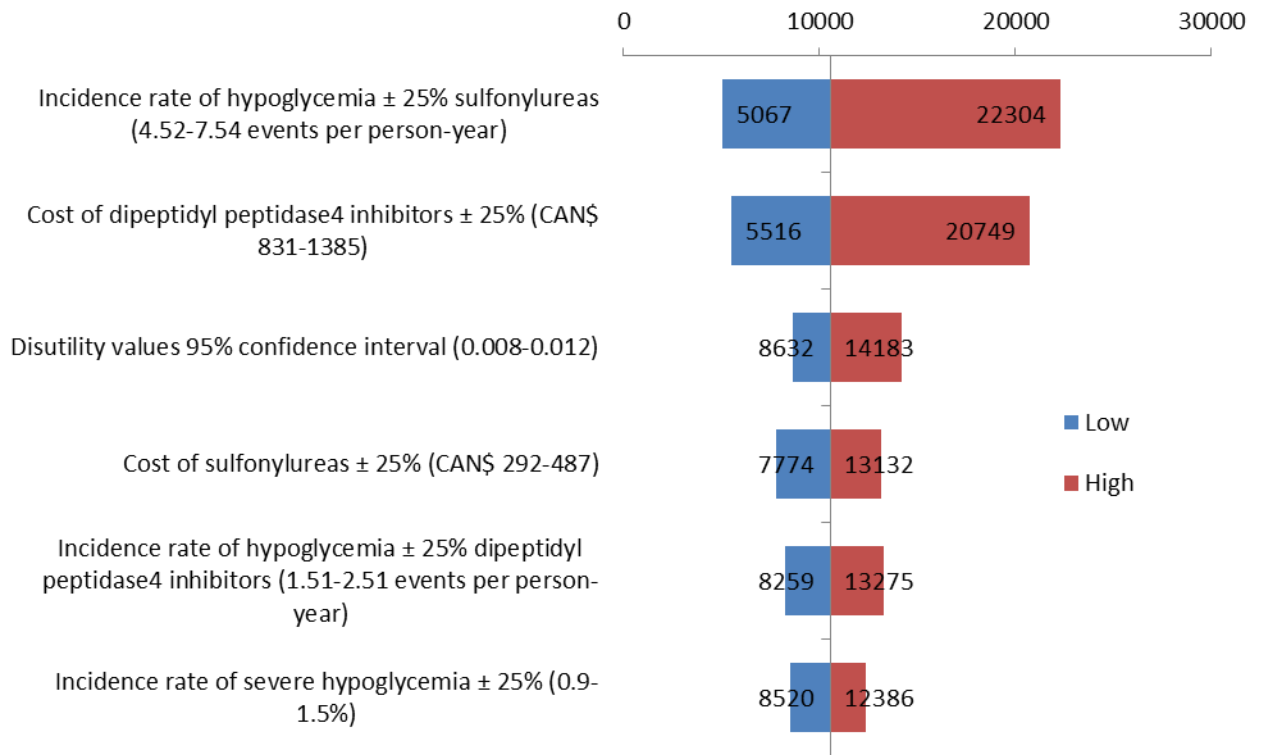

G.

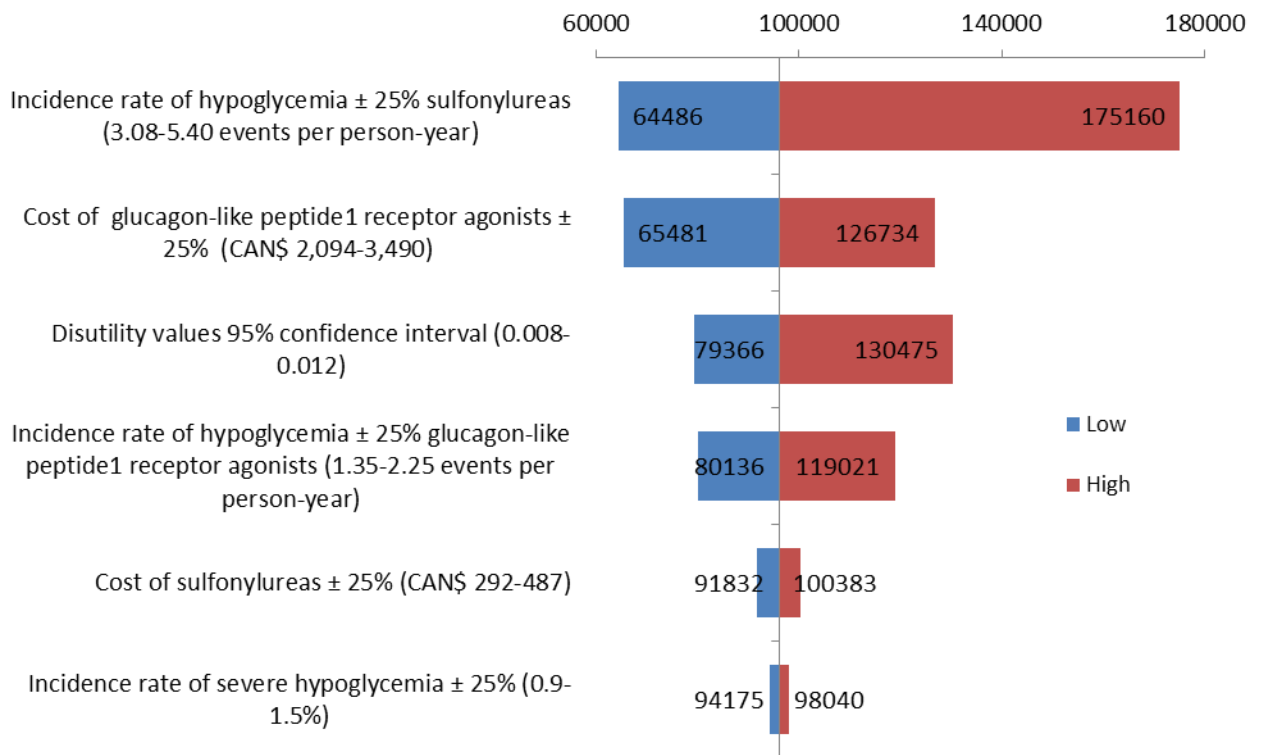

H.

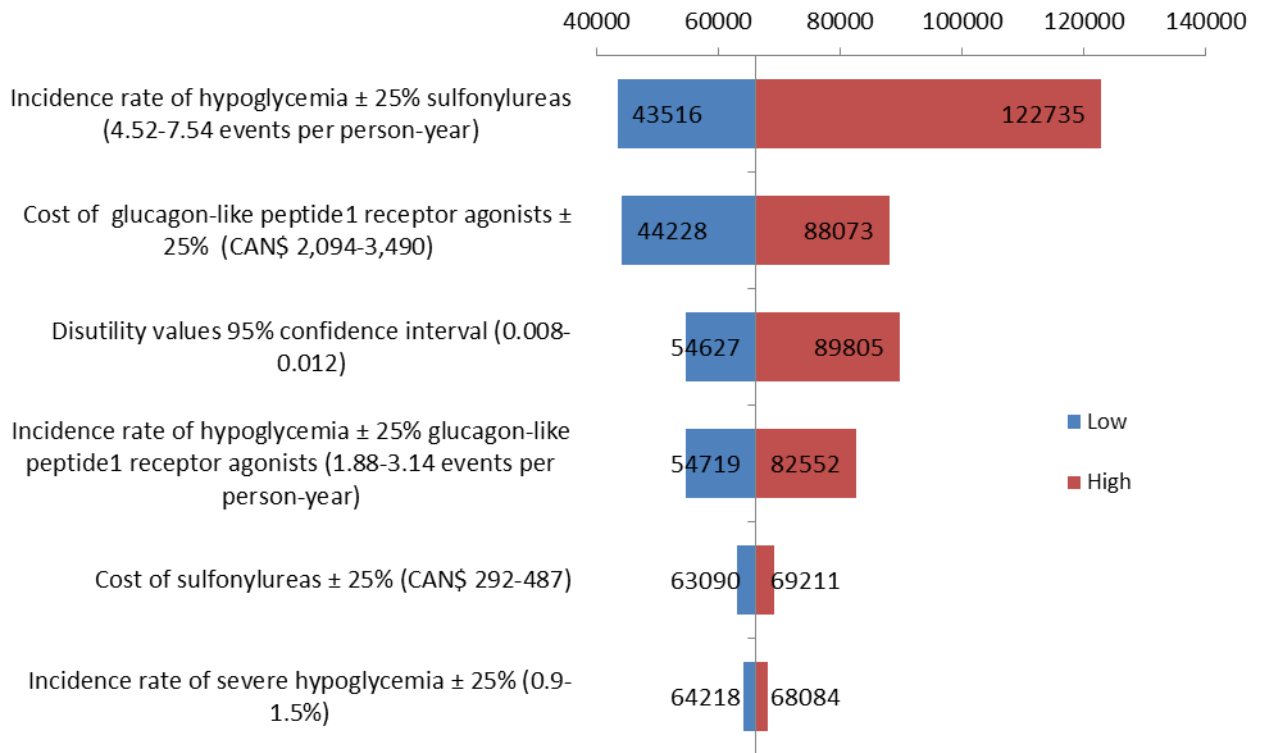

Supplement: S1 Fig — (A) For type 2 diabetic adults aged 65–79 years using dipeptidyl peptidase4 inhibitors relative to sulfonylureas in the U.S. The incremental cost per quality-adjusted life-year gained in the base-case was U.S.$61,342. (B) For type 2 diabetic adults aged 80 years and older using dipeptidyl peptidase4 inhibitors relative to sulfonylureas in the U.S. The incremental cost per quality-adjusted life-year gained in the base-case was U.S.$41,746. (C) For type 2 diabetic adults aged 65–79 years using glucagon-like peptide1 receptor agonists relative to sulfonylureas in the U.S. The incremental cost per quality-adjusted life-year gained in the base-case was U.S.$71,193. (D) For type 2 diabetic adults aged 80 years and older using glucagon-like peptide1 receptor agonists relative to sulfonylureas in the U.S. The incremental cost per quality-adjusted life-year gained in the base-case was U.S.$48,796. (E) For type 2 diabetic adults aged 65–79 years using dipeptidyl peptidase4 inhibitors relative to sulfonylureas in Canada. The incremental cost per quality-adjusted life-year gained in the base-case was CAN$18,378. (F) For type 2 diabetic adults aged 80 years and older using dipeptidyl peptidase4 inhibitors relative to sulfonylureas in Canada. The incremental cost per quality-adjusted life-year gained in the base-case was CAN$10,539. (G) For type 2 diabetic adults aged 65–79 years using glucagon-like peptide1 receptor agonists relative to sulfonylureas in Canada. The incremental cost per quality-adjusted life-year gained in the base-case was CAN$96,201.(H) For type 2 diabetic adults aged 80 years and older using glucagon-like peptide1 receptor agonists relative to sulfonylureas in Canada. The incremental cost per quality-adjusted life-year gained in the base-case was CAN$66,244. (PDF) [file pone.0162951.s001.pdf]

A.

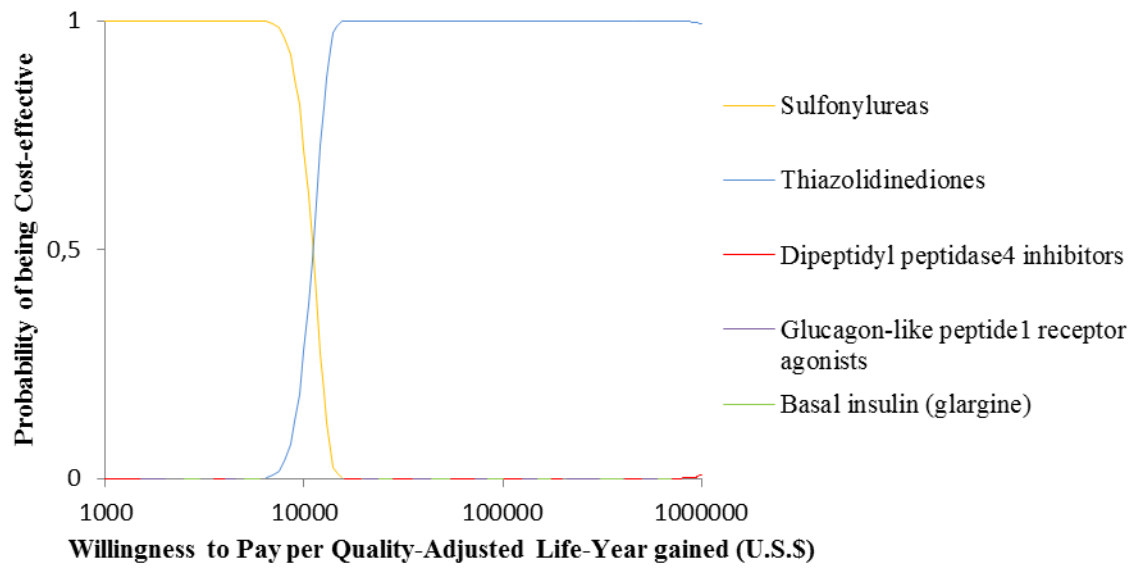

B.

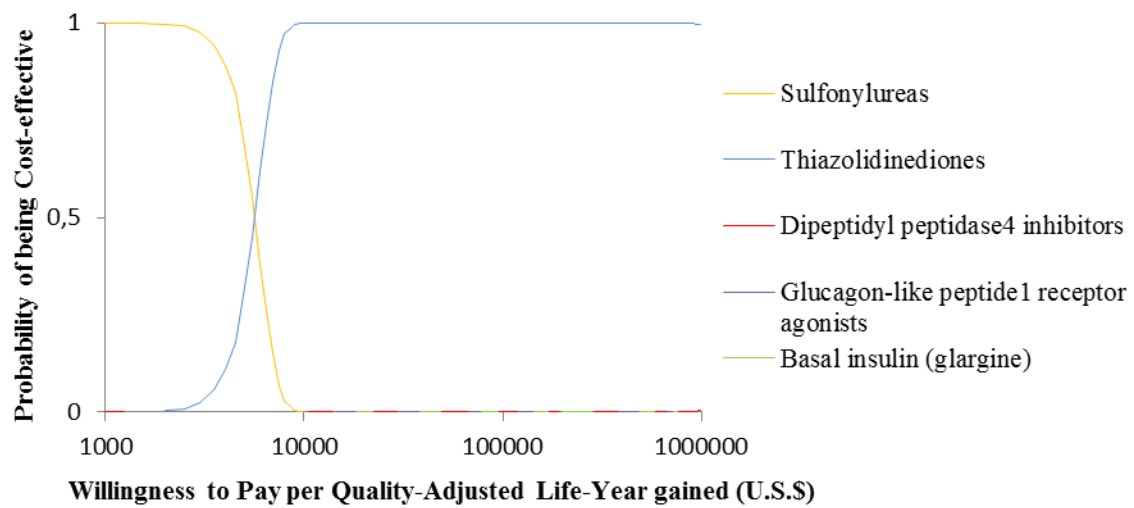

C.

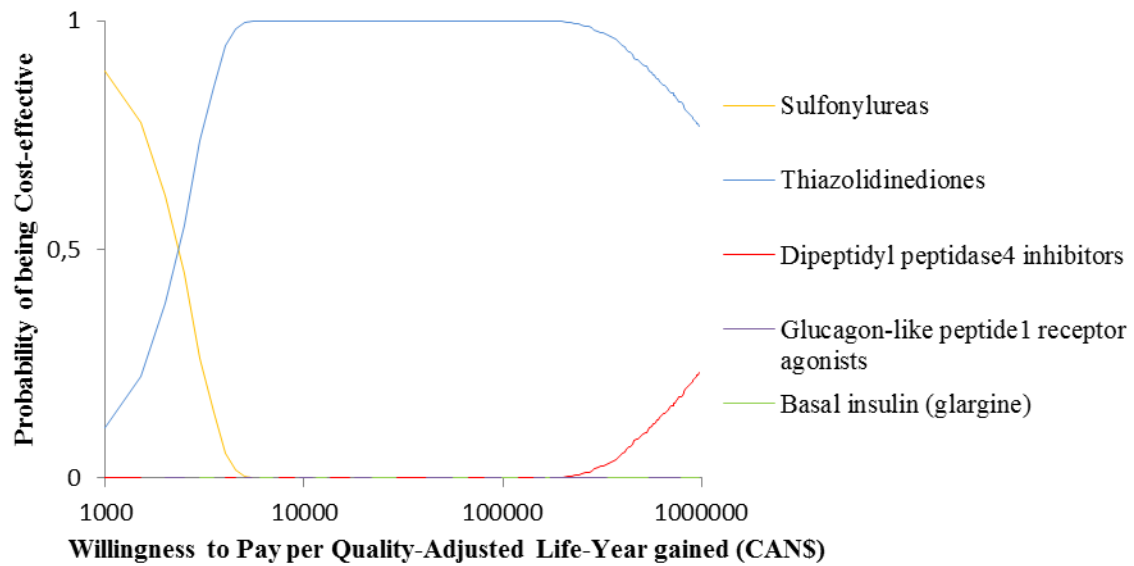

D.

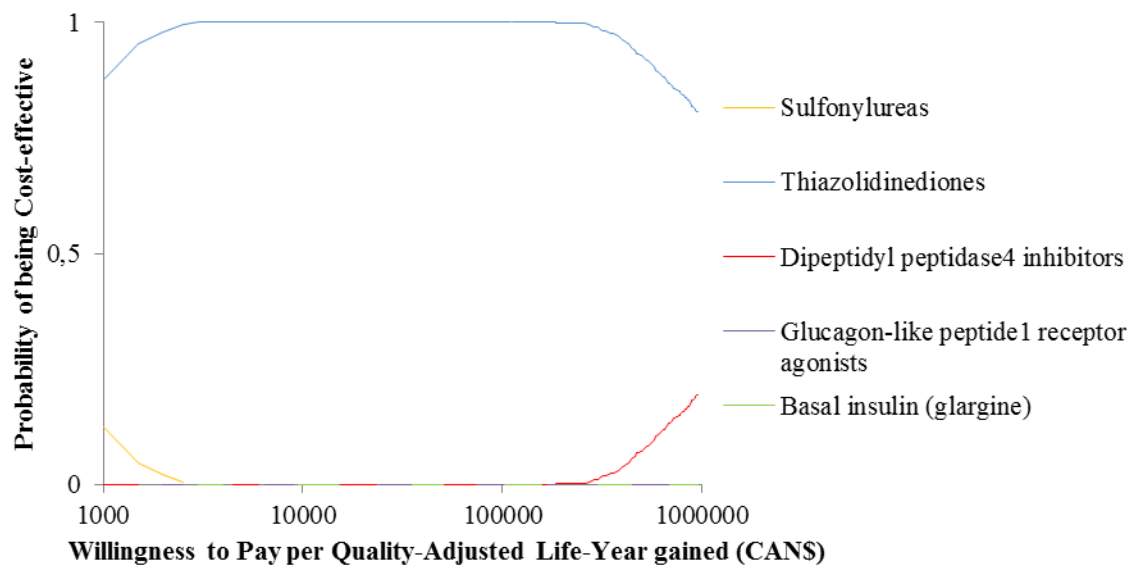

Supplement: S2 Fig — (A) For type 2 diabetic adults aged 65–79 years in the U.S. (B) For type 2 diabetic adults aged 80 years and older in the U.S. (C) For type 2 diabetic adults aged 65–79 years in Canada. (D) For type 2 diabetic adults aged 80 years and older in Canada. (PDF) [file pone.0162951.s002.pdf]
