# Supplementary material for: Preventing Unnecessary Costs of Drug-Induced Hypoglycemia in Older Adults with Type 2 Diabetes in the United States and Canada
Source: PLoS One. 2016 Sep 20;11(9):e0162951. doi: 10.1371/journal.pone.0162951 (PMC5029920; doi:10.1371/journal.pone.0162951)
Supplement: S1 Table — (DOCX) [file pone.0162951.s003.docx]

**S1 Table**

**Cost of healthcare resource use and glucose-lowering therapy**

|  | Unit | Cost  (2015 U.S.$) | Assumptions/Sources with references | Cost  (2015 CAN$) | Assumptions/Sources with references |
| --- | --- | --- | --- | --- | --- |
| **Healthcare resource** |  |  |  |  |  |
| *Primary/secondary care* |  |  |  |  |  |
| General practitioner visit | 1 | 55.86 | 2015 U.S. Medicare Physician Fee Schedule (99213, physician visit for medium, established patient) [1] | 77.20 | 2015 Ontario Schedule of Benefits for Physician Services (K132, physician visit for patient aged 65 years and older) [8] |
| Nurse practitioner visit | 1 | 47.48 | 85% of 99213 [1] | 65.62 | 85% of K132 [8] |
| Outpatient | 1 | 332.05^a^ | U.S. insurance claims data [6] | 300.70 | 2015 Ontario Schedule of Benefits for Physician Services (A150, comprehensive endocrinology consultation [8] |
| Emergency room visit | 1 | 1,163.17^*^ | Direct medical cost for hypoglycemia-related emergency room visit in the U.S. (including physician fees) [7] | 1,461.43 | No accessible data in Canada; costs converted to 2015 CAN$ from original 2015 U.S. cost data [7,13] |
| Inpatient with emergency room | 1 | 16,794.36^*^ | Direct medical cost for hypoglycemia-related emergency room visit in the U.S. (including physician fees) [7] | 21,103.34 | “ |
| Ambulance | 1 | 360.86 | 2015 U.S. Medicare Ambulance Fee Schedule, base rate for an ambulance (Basic Life Support – Emergency A0429) averaged across state localities [2] | 332.35 | 2015 average ground ambulance service fee in Alberta [11] |
| *Other* |  |  |  |  |  |
| Blood glucose, quantitative assay | 1 | 5.34 | 2015 U.S. Medicare Clinical Laboratory Fee Schedule (82947) [3] | 5.00 | 2015 Ontario Schedule of Benefits for Laboratory Services (L111) [9] |
| Self-monitoring blood glucose test | 1 | 0.81 | 2015 US Medicare Durable Medical Equipment, Prosthetics, orthotics, and supplies Fee Schedule (A4253, 0.71 per strip + A4259, 0.10 per lancet) [4] | 0.75 | 2015 Ontario drug benefit formulary/Comparative drug index (0.72 per strip); 2015 manufacturer list price (0.02 per lancet) [10,12] |
| Glucagon 1 mg | 1 | 152.12 | National average drug acquisition cost of a Glucagon Emergency Kit vial [5] | 79.27 | 2015 Ontario drug benefit formulary/Comparative drug index [10] |
| **Therapy** |  |  |  |  |  |
| *Medication* |  |  |  |  |  |
| Metformin | Daily | 0.03 | 1,000 mg; 100% of generic use [5] | 0.09 | 1,000 mg; 100% of generic use [10] |
|  |  |  |  |  |  |
| Sulfonylureas | 1-day | 0.13 | Glyburide modified release 1.25 mg, glimepiride 2 mg, glipizide modified release 10 mg; 100% of generics use [5] | 0.20 | Glyburide 2.5 mg, glimepiride 2 mg, gliclazide modified release 60 mg; 100% of generics use [10] |
| Dipeptidyl peptidase4 inhibitors | 1-day | 10.67 | Lanagliptin 5 mg, saxagliptin 2.5 mg, sitagliptin 50 mg [5] | 2.64 | Lanagliptin 5 mg, saxagliptin 2.5 mg, sitagliptin 50 mg [10] |
| Thiazolidinediones | 1-day | 2.11 | Pioglitazone 30 mg (100% of generic use), rosiglitazone 4 mg [5] | 1.55 | Pioglitazone 30 mg (100% of generic use), rosiglitazone 4 mg |
| Glucagon-like peptide1 receptor agonists | 1-day | 7.59 | Liraglutide 0.6 mg, exenatide 10 mcg [5] | 6.85 | Liraglutide 0.6 mg [22] |
| Basal insulin | 1-day | 3.21 | 40 International Units [5] | 8.23 | 40 International Units [10] |
| *Other* |  |  |  |  |  |
| Dispensing fee | Monthly | 10.81 | 2015 Medicaid Prescription Reimbursement Information by State [18] | 2.33 | 2015 Ontario Drug Benefit Program [20] |
| Needles (30 gauge) | 1 | 0.53 | 2015 wholesale acquisition cost [19] | 0.47 | 2015 Ontario Assistive Devices program for seniors [23] |
| Home blood glucose monitor | Annual | 7.41 | 2015 U.S. Medicare Durable Medical Equipment, Prosthetics, orthotics, and supplies Fee Schedule (E0607, rent) [4] | 11.25 | 2015 Ontario Monitoring for Health Program [24] |

^*^ Original costs were inflated using Consumer Price Index inflation calculator from the Bureau Labor of Statistics, available at <http://www.bls.gov/data/inflation_calculator.htm>
