# Supplementary material for: Preventing Unnecessary Costs of Drug-Induced Hypoglycemia in Older Adults with Type 2 Diabetes in the United States and Canada
Source: PLoS One. 2016 Sep 20;11(9):e0162951. doi: 10.1371/journal.pone.0162951 (PMC5029920; doi:10.1371/journal.pone.0162951)
Supplement: S2 Table — (DOCX) [file pone.0162951.s004.docx]

**S2 Table**

**Utility, disutility and frequency of events data used in the model**

|  | Values (95% confidence interval) or frequency | Reference |
| --- | --- | --- |
| Utility for uncomplicated diabetes | 0.844 (0.839 - 0.848) | [25] |
| Disutility for daytime* mild hypoglycemic event | 0.005 (0.003 - 0.006) | [25] |
| Disutility for nocturnal mild hypoglycemic event | 0.007 (0.005 - 0.010) | [25] |
| Proportion of daytime* versus nighttime events for mild hypoglycemia | 0.75 | [26] |
| Disutility for daytime* moderate or severe hypoglycemic event | 0.060 (0.051 - 0.069) | [25] |
| Disutility for nocturnal moderate or severe hypoglycemic event | 0.078 (0.067 - 0.089) | [25] |
| Proportion of daytime* versus nighttime events for moderate/severe hypoglycemia | 0.60 | [27] |

^*^ Daytime is 08:00-00:00
