# Supplementary material for: Preventing Unnecessary Costs of Drug-Induced Hypoglycemia in Older Adults with Type 2 Diabetes in the United States and Canada
Source: PLoS One. 2016 Sep 20;11(9):e0162951. doi: 10.1371/journal.pone.0162951 (PMC5029920; doi:10.1371/journal.pone.0162951)
Supplement: S3 Table — (DOCX) [file pone.0162951.s005.docx]

| **S3 Table**  **Potential annual savings from disinvesting in insulin and sulfonylurea prescriptions in older type 2 diabetic adults attaining very tight glycemic control** | | | | | | |
| --- | --- | --- | --- | --- | --- | --- |
|  | U.S. (2015 U.S.$) | | | Canada (2015 CAN$) | | |
|  | Insulin discontinuation | Sulfonylureas discontinuation | Total | Insulin discontinuation | Sulfonylureas discontinuation | Total |
| No drug replacement, 100% | 623,796,800 | 583,425,333 | 1,207,222,133 | 89,247,725 | 70,546,131 | 159,793,856 |
| Replacement with metformin, 100% | 420,940,667 | 250,082,067 | 671,022,734 | 76,727,349 | 41,749,267 | 118,476,616 |
| Replacement with metformin/thiazolidinediones, 50%/50% | 317,724,000 | 12,683,733 | 330,407,733 | 33,774,845 | 18,160,079 | 51,934,925 |
