# Supplementary material for: Preventing Unnecessary Costs of Drug-Induced Hypoglycemia in Older Adults with Type 2 Diabetes in the United States and Canada
Source: PLoS One. 2016 Sep 20;11(9):e0162951. doi: 10.1371/journal.pone.0162951 (PMC5029920; doi:10.1371/journal.pone.0162951)
Supplement: S1 Text — (DOCX) [file pone.0162951.s006.docx]

**Supporting information**

**Cost of healthcare resource use and glucose-lowering therapy in the United States and Canada**

For U.S. healthcare resources, costs were obtained from the Center for Medicare Services files [1-5] and from economic national data in type 2 diabetic adults for outpatient visits [6], emergency room visits [7], and hospitalizations for hypoglycemia [7]. For Canadian healthcare resources, costs were obtained from the Ontario Ministry of Health and Long-Term Care [8-10], from Alberta Ministry of Health for ambulance fees [11], and from manufacturer list price for needles used during blood glucose monitoring [12]. There is no accessible data for the cost of emergency room visit or hospitalization for hypoglycemia in Canada. Therefore, these costs were converted to 2015 CAN$ from original 2015 U.S. Medicare cost data using an exchange rate of 1.2566 on July 3, 2015 [13].

For the different medication classes, the total U.S. or Canadian therapy cost included medication acquisition costs, testing supplies cost (strips, needles, and blood sugar monitor) as well as the cost of needles for injectable drugs (basal insulin and glucagon-like peptide1 receptor agonists). Average medication cost was estimated using an equal proportion of use of each drug in the case of several drugs being available in a given medication class. For drugs that did not require dose reduction in older adults or renal impairment (except for a glomerular function rate below 15 ml/mn), namely gliclazide, glimepiride, glipizide, thiazolidinediones and insulin [14], we assumed daily doses as specified by the World Health Organization [15]. For other drugs, the lowest dose was considered. Only glyburide, glimepiride, and glipizide (in the U.S.) or gliclazide (in Canada) were taken into account for the sulfonylureas cost estimation.

In the U.S., glucose-lowering medications and needles for administration of insulin or glucagon-like peptide1 receptor agonists are covered by Medicare Part D [16]. In the 2015 Medicare model standard plan, the Medicare prescription drug plan pays 75% of the medication costs up to U.S.$2,640 (the initial coverage limit U.S.$2,960 minus the U.S.$320 deductible) [16]. In plan year 2015, after the initial coverage limit is reached, Medicare Part D pays 35% of generic medication costs in the donut hole and the brand-name manufacturer will pay 55% of brand-name medication costs in the donut hole, even if the plan’s coverage states that it has no gap coverage [16]. Prices were obtained for each drug from the National Average Drug Acquisition Cost file (weekly file as of 06/10/2015) [5] as reflecting the actual average cost of a drug [17]. An average monthly dispensing fee of U.S.$10.81 was added to estimate the average annual medication cost [18]. The price for 30 gauge needles was obtained from published wholesale acquisition costs in the U.S. [19]. Testing supplies are covered by Medicare Part B with fees obtained from the Center for Medicare Services files [4].

In Canada, we obtained unit drug costs from the Ontario Drug Benefit Program for patients aged 65 years and older [20]. Accordingly, a 10% markup and CAN$7 pharmacy fee per 90-day were added and an average monthly co-payment of CAN$4.06 was deducted (minimum $2, maximum $6.11) to calculate the annual medication cost [20]. For glucagon-like peptide1 receptor agonists, only liraglutide is covered through one Canadian/provincial public program [21]. Thus, the liraglutide price was derived from the Régie de l’Assurance Maladie du Québec file [22]. For needles, seniors receive an annual grant of CAN$170 through the Assistive Devices Program [23]. The number of blood glucose test strips permitted in the Ontario Drug Benefit Program is 3,000 for insulin, 400 for sulfonylureas, and 200 for other diabetic medications [10]. Needles for blood glucose monitoring and blood glucose meter are reimbursed at 75% in diabetic adults aged 65 years and older through the Ontario Monitoring for Health Program, funded by the Ontario Ministry of Health and Long-Term Care [24].

**S1 Table**

**Cost of healthcare resource use and glucose-lowering therapy**

|  | Unit | Cost  (2015 U.S.$) | Assumptions/Sources with references | Cost  (2015 CAN$) | Assumptions/Sources with references |
| --- | --- | --- | --- | --- | --- |
| **Healthcare resource** |  |  |  |  |  |
| *Primary/secondary care* |  |  |  |  |  |
| General practitioner visit | 1 | 55.86 | 2015 U.S. Medicare Physician Fee Schedule (99213, physician visit for medium, established patient) [1] | 77.20 | 2015 Ontario Schedule of Benefits for Physician Services (K132, physician visit for patient aged 65 years and older) [8] |
| Nurse practitioner visit | 1 | 47.48 | 85% of 99213 [1] | 65.62 | 85% of K132 [8] |
| Outpatient | 1 | 332.05^a^ | U.S. insurance claims data [6] | 300.70 | 2015 Ontario Schedule of Benefits for Physician Services (A150, comprehensive endocrinology consultation [8] |
| Emergency room visit | 1 | 1,163.17^*^ | Direct medical cost for hypoglycemia-related emergency room visit in the U.S. (including physician fees) [7] | 1,461.43 | No accessible data in Canada; costs converted to 2015 CAN$ from original 2015 U.S. cost data [7,13] |
| Inpatient with emergency room | 1 | 16,794.36^*^ | Direct medical cost for hypoglycemia-related emergency room visit in the U.S. (including physician fees) [7] | 21,103.34 | “ |
| Ambulance | 1 | 360.86 | 2015 U.S. Medicare Ambulance Fee Schedule, base rate for an ambulance (Basic Life Support – Emergency A0429) averaged across state localities [2] | 332.35 | 2015 average ground ambulance service fee in Alberta [11] |
| *Other* |  |  |  |  |  |
| Blood glucose, quantitative assay | 1 | 5.34 | 2015 U.S. Medicare Clinical Laboratory Fee Schedule (82947) [3] | 5.00 | 2015 Ontario Schedule of Benefits for Laboratory Services (L111) [9] |
| Self-monitoring blood glucose test | 1 | 0.81 | 2015 US Medicare Durable Medical Equipment, Prosthetics, orthotics, and supplies Fee Schedule (A4253, 0.71 per strip + A4259, 0.10 per lancet) [4] | 0.75 | 2015 Ontario drug benefit formulary/Comparative drug index (0.72 per strip); 2015 manufacturer list price (0.02 per lancet) [10,12] |
| Glucagon 1 mg | 1 | 152.12 | National average drug acquisition cost of a Glucagon Emergency Kit vial [5] | 79.27 | 2015 Ontario drug benefit formulary/Comparative drug index [10] |
| **Therapy** |  |  |  |  |  |
| *Medication* |  |  |  |  |  |
| Metformin | Daily | 0.03 | 1,000 mg; 100% of generic use [5] | 0.09 | 1,000 mg; 100% of generic use [10] |
|  |  |  |  |  |  |
| Sulfonylureas | 1-day | 0.13 | Glyburide modified release 1.25 mg, glimepiride 2 mg, glipizide modified release 10 mg; 100% of generics use [5] | 0.20 | Glyburide 2.5 mg, glimepiride 2 mg, gliclazide modified release 60 mg; 100% of generics use [10] |
| Dipeptidyl peptidase4 inhibitors | 1-day | 10.67 | Lanagliptin 5 mg, saxagliptin 2.5 mg, sitagliptin 50 mg [5] | 2.64 | Lanagliptin 5 mg, saxagliptin 2.5 mg, sitagliptin 50 mg [10] |
| Thiazolidinediones | 1-day | 2.11 | Pioglitazone 30 mg (100% of generic use), rosiglitazone 4 mg [5] | 1.55 | Pioglitazone 30 mg (100% of generic use), rosiglitazone 4 mg |
| Glucagon-like peptide1 receptor agonists | 1-day | 7.59 | Liraglutide 0.6 mg, exenatide 10 mcg [5] | 6.85 | Liraglutide 0.6 mg [22] |
| Basal insulin | 1-day | 3.21 | 40 International Units [5] | 8.23 | 40 International Units [10] |
| *Other* |  |  |  |  |  |
| Dispensing fee | Monthly | 10.81 | 2015 Medicaid Prescription Reimbursement Information by State [18] | 2.33 | 2015 Ontario Drug Benefit Program [20] |
| Needles (30 gauge) | 1 | 0.53 | 2015 wholesale acquisition cost [19] | 0.47 | 2015 Ontario Assistive Devices program for seniors [23] |
| Home blood glucose monitor | Annual | 7.41 | 2015 U.S. Medicare Durable Medical Equipment, Prosthetics, orthotics, and supplies Fee Schedule (E0607, rent) [4] | 11.25 | 2015 Ontario Monitoring for Health Program [24] |

^*^ Original costs were inflated using Consumer Price Index inflation calculator from the Bureau Labor of Statistics, available at <http://www.bls.gov/data/inflation_calculator.htm>

**S2 Table**

**Utility, disutility and frequency of events data used in the model**

|  | Values (95% confidence interval) or frequency | Reference |
| --- | --- | --- |
| Utility for uncomplicated diabetes | 0.844 (0.839 - 0.848) | [25] |
| Disutility for daytime* mild hypoglycemic event | 0.005 (0.003 - 0.006) | [25] |
| Disutility for nocturnal mild hypoglycemic event | 0.007 (0.005 - 0.010) | [25] |
| Proportion of daytime* versus nighttime events for mild hypoglycemia | 0.75 | [26] |
| Disutility for daytime* moderate or severe hypoglycemic event | 0.060 (0.051 - 0.069) | [25] |
| Disutility for nocturnal moderate or severe hypoglycemic event | 0.078 (0.067 - 0.089) | [25] |
| Proportion of daytime* versus nighttime events for moderate/severe hypoglycemia | 0.60 | [27] |

^*^ Daytime is 08:00-00:00

| **S3 Table**  **Potential annual savings from disinvesting in insulin and sulfonylurea prescriptions in older type 2 diabetic adults attaining very tight glycemic control** | | | | | | |
| --- | --- | --- | --- | --- | --- | --- |
|  | U.S. (2015 U.S.$) | | | Canada (2015 CAN$) | | |
|  | Insulin discontinuation | Sulfonylureas discontinuation | Total | Insulin discontinuation | Sulfonylureas discontinuation | Total |
| No drug replacement, 100% | 623,796,800 | 583,425,333 | 1,207,222,133 | 89,247,725 | 70,546,131 | 159,793,856 |
| Replacement with metformin, 100% | 420,940,667 | 250,082,067 | 671,022,734 | 76,727,349 | 41,749,267 | 118,476,616 |
| Replacement with metformin/thiazolidinediones, 50%/50% | 317,724,000 | 12,683,733 | 330,407,733 | 33,774,845 | 18,160,079 | 51,934,925 |

**Supplementary references**

1. Centers for Medicare and Medicaid Services. Physician fee schedule 2015 (online). Available at https://www.cms.gov/Medicare/Medicare-Fee-for-Service-Payment/PhysicianFeeSched/index.html?redirect=/PhysicianFeeSched/. Accessed December 28, 2015.
2. Centers for Medicare and Medicaid Services. Ambulance fee schedule 2015 (online). Available at https://www.cms.gov/Medicare/Medicare-Fee-for-Service-Payment/AmbulanceFeeSchedule/http://www.cms.gov/Medicare/Medicare-Fee-for-Service-Payment/AmbulanceFeeSchedule/afspuf.html. Accessed December 28, 2015.
3. Centers for Medicare and Medicaid Services. Clinical Laboratory fee schedule 2015 (online). Available from https://www.cms.gov/Medicare/Medicare-Fee-for-Service-Payment/ClinicalLabFeeSched/http://www.cms.gov/Medicare/Medicare-Fee-for-Service-Payment/ClinicalLabFeeSchedule/clinlab.html. Accessed December 28, 2015.
4. Centers for Medicare and Medicaid Services. Durable Medical Equipment, Prosthetics, orthotics, and supplies fee schedule. Available 2015(online). Available at <http://www.cms.gov/Medicare/Medicare-Fee-for-Service-Payment/DMEPOSFeeSched/DMEPOS-Fee-Schedule-Items/DME15-A.html?DLPage=1&DLEntries=10&DLSort=2&DLSortDir=descending>. Accessed December 28, 2015.
5. Centers for Medicare and Medicaid Services. National Average Drug Acquisition Cost (weekly file as of 06/10/2015) 2015 (online). Available at <http://www.medicaid.gov/Medicaid-CHIP-Program-Information/By-Topics/Benefits/Prescription-Drugs/Pharmacy-Pricing.html>. Accessed December 11, 2015.
6. Curkendall S, Zhang B, Oh KS et al. Incidence and cost of hypoglycemia among patients with type 2 diabetes in the United States: Analysis of a healthcare Insurance Database. JCOM 2011;18;455-462.
7. Ward A, Alvarez P, Vo L, Martin S. [Direct medical costs of complications of diabetes in the United States: estimates for event-year and annual state costs (USD 2012).](http://www.ncbi.nlm.nih.gov/pubmed/24410011) J Med Econ 2014;17:176-183.
8. Ontario Ministry of Health and Long-term Care. Schedules of benefits for physician services 2015 (online). Available at <http://www.health.gov.on.ca/english/providers/program/ohip/sob/physserv/sob_master11062015.pdf>. Accessed December 3, 2015.
9. Ontario Ministry of Health and Long-term Care. Schedules of benefits for laboratory services 2015 (online). Available at <http://www.health.gov.on.ca/english/providers/program/ohip/sob/physserv/sob_master11062015.pdf>. Accessed December 3, 2015.
10. Ontario Ministry of Health and Long-term Care. Ontario drug benefit formulary/Comparative drug index. Edition 42, 2015 (online). Available at [http://www.health.gov.on.ca/en/pro/programs/drugs/formulary42/edition_42.pdf.](http://www.health.gov.on.ca/en/pro/programs/drugs/formulary42/edition_42.pdf.%20)  Accessed December 3, 2015.
11. Health Alberta. Who pays for ambulance services? 2015 (online). Available from http://www.health.alberta.ca/services/EHS-who-pays.html. Accessed July 3, 2015.
12. Mc Kesson. Manufacturer list price for lancets 2015 (online). Available at <http://www.mckesson.com/pharmacies/independent-retail/front-end-purchasing-programs/sunmark-private-brand/>. Accessed December 3, 2015.
13. Bank of Canada: 10-Year currency converter 2015 (online). Available at <http://www.bankofcanada.ca/rates/exchange/10-year-converter/>. Accessed December 3, 2015.
14. U.S. Food and Drug Administration. Drug approvals and databases 2015 (online). Available at <http://www.fda.gov/Drugs/InformationOnDrugs/default.htm>. Accessed December 28, 2015.
15. World Health Organization. Collaborating centre for drugs statistics methodology. ATC/DDD index, 2015 (online). Available at <http://www.whocc.no/atc_ddd_index/>. Accessed December 28, 2015.
16. Medicare Part D RX Plans, 2015 (online). Available at <http://www.q1medicare.com/PartD-The-2015-Medicare-Part-D-Outlook.php>. Accessed December 28, 2015.
17. Mullins CD, Seal B, Seoane-Vazquez E, et al. [Good research practices for measuring drug costs in cost-effectiveness analyses: Medicare, Medicaid and other US government payers perspectives: the ISPOR Drug Cost Task Force report--Part IV.](http://www.ncbi.nlm.nih.gov/pubmed/19807903) Value Health 2010;13:18-24.
18. Centers for Medicare and Medicaid Services. Medicaid Prescription Reimbursement Information by State, 2014 (online). Available at <http://www.medicaid.gov/Medicaid-CHIP-Program-Information/By-Topics/Benefits/Prescription-Drugs/Downloads/reimbursementchart-2Q2013.pdf>. Accessed December 28, 2015.
19. MEDI-Span. Price Rx. Walter Kluwers Health, 2015 (online). Available at [http://www.medispan.com/drug-pricing-analysis-pricerx.aspx. Accessed December 11](http://www.medispan.com/drug-pricing-analysis-pricerx.aspx.%20Accessed%20December%2011), 2015.
20. Ontario Ministry of Health and Long-term Care. Ontario Drug Benefit Program for seniors, 2015 (online). Available at <http://www.health.gov.on.ca/en/public/programs/drugs/programs/odb/opdp_after65.aspx>. Accessed December 3, 2015.
21. Canada drug coverage. A guide to reimbursement, 2015 (online). Available at <http://www.drugcoverage.ca/en-ca/search-results.aspx?ID=7451>. Accessed December 3, 2015.
22. Régie de l’Assurance Maladie du Québec. Liste des médicaments, 2015 (online). Available at https://www.prod.ramq.gouv.qc.ca/DPI/PO/Commun/PDF/Liste_Med/Liste_Med/liste_med_2015_06_01_fr.pdf. Accessed December 3, 2015.
23. Ontario Ministry of Health and Long-term Care. Assistive Devices Program, 2015 (online). Available at http://www.health.gov.on.ca/en/public/programs/adp/publications/diabetic.aspx. Accessed December 3, 2015.
24. Canadian Diabetes Association. Ontario Monitoring for Health Program, 2015 (online). Available at <https://www.diabetes.ca/CDA/media/documents/programs-and-support/in-your-community/Regional%20events-programs/pharmacist-information-monitoring-for-health-program.pdf>. Accessed December 3, 2015.
25. Evans M, Khunti K, Mamdani M et al. Health-related quality of life associated with daytime and nocturnal hypoglycaemic events: a time trade-off survey in five countries. Health Qual Life Outcomes 2013;11:90.
26. Brod M, Christensen T, Thomsen TL, Bushnell DM. The impact of non-severe hypoglycemic events on work productivity and diabetes management. Value Health 2011;14:665-671.
27. Gold AE, MacLeod KM, Frier BM. [Frequency of severe hypoglycemia in patients with type I diabetes with impaired awareness of hypoglycemia.](http://www.ncbi.nlm.nih.gov/pubmed/7924780) Diabetes Care 1994;17:697-703.
